# The Field’s mass shooting: emergency medical services response

**DOI:** 10.1186/s13049-023-01140-7

**Published:** 2023-11-02

**Authors:** Peter Martin Hansen, Søren Mikkelsen, Henrik Alstrøm, Anders Damm-Hejmdal, Marius Rehn, Peter Anthony Berlac

**Affiliations:** 1 The Mobile Emergency Care Unit, Department of Anesthesiology and Intensive Care, Odense University Hospital Svendborg, Svendborg, Denmark; 2 Danish Air Ambulance, Aarhus, Denmark; 3 The Prehospital Research Unit, Region of Southern Denmark, Odense University Hospital, Odense, Denmark; 4 The Mobile Emergency Care Unit, Department of Anesthesiology and Intensive Care, Odense University Hospital, Odense, Denmark; 5 Department of Regional Health Research, University of Southern Denmark, Odense, Denmark; 6 Department of Anesthesiology and Intensive Care, Herlev and Gentofte Hospital, Herlev, Denmark; 7 Copenhagen Emergency Medical Services, Ballerup, Denmark; 8 Dept. of Research and Development, Norwegian Air Ambulance Foundation, Oslo, Norway; 9 Air Ambulance Department, Division of Prehospital Services, Oslo University Hospital, Oslo, Norway; 10 Institute of Clinical Medicine, University of Oslo, Oslo, Norway; 11 Department of Anesthesiology and Intensive Care, Hvidovre and Amager Hospital, Hvidovre, Denmark

**Keywords:** Major incident, Disaster, Mass shooting, Mass casualty, Management and leadership

## Abstract

**Background:**

Major incidents (MI) happen infrequently in Scandinavia and mass shootings are even less frequently occurring. Case reports and research are called for, as literature is scarce. On 3rd July 2022, a mass shooting took place at the shopping mall Field’s in Copenhagen, Denmark. Three people were killed and seven injured by a gunman, firing a rifle inside the mall. A further 21 people suffered minor injuries during the evacuation of the mall. In this case report, we describe the emergency medical services (EMS) incident response and evaluate the EMS´ adherence to the MI management guidelines to identify possible areas of improvement.

**Case presentation:**

Forty-eight EMS units including five Tactical Emergency Medical Service teams were dispatched to the incident. Four critically injured patients were taken to two trauma hospitals. The deceased patients were declared dead at the scene and remained there for the sake of the investigation. A total of 24 patients with less severe and minor injuries were treated at four different hospitals in connection with the attack. The ambulance resources were inherently limited in the initial phase of the MI, mandating improvisation in medical incident command. Though challenged, Command and Control, Safety, Communication, Assessment, Triage, Treatment, Transport (CSCATTT) principles were followed.

**Conclusions:**

The EMS response generally adhered to national guidelines for MI. The activation of EMS and the hospital preparedness program was relevant. Important findings were communication shortcomings; inherent lack of readily available ambulance resources in the initial critical phase; uncertainty regarding the number of perpetrators; uncertainty regarding number of casualties and social media rumors that unnecessarily hampered and prolonged the response. The incident command had to use non-standard measures to mitigate potential challenges.

**Supplementary Information:**

The online version contains supplementary material available at 10.1186/s13049-023-01140-7.

## Background

Major incidents (MI), defined by the need for mobilization of extraordinary resources [1], happen infrequently and epidemiology literature is scarce [2] and of heterogeneous quality. MI in the form of mass shootings in Scandinavia have occurred occasionally in Norway and Finland [3, 4] and are subjected to extensive media coverage [5]. Therefore, and obviously with the purpose of saving lives, the importance of MI preparedness in the emergency medical services (EMS) remains substantive.

On Sunday 3rd July 2022, a mass shooting took place in the shopping mall Field’s in Copenhagen, Denmark. Three people were killed, and seven people injured from gunshots, four of these seriously. Twenty-one people sustained injuries not related to gunfire during the evacuation or while hiding inside the mall. In all, 28 patients were treated at four different hospitals in the Capital Region.

The Field’s shooting elicited a massive EMS response. In this case report, we aim to provide a detailed description of the prehospital EMS response to the incident. We also aim to evaluate the adherence to guidelines for future MI management purposes.

## Case presentation

### Danish emergency medical services

The Danish national distress number 1–1-2 provides one point of entry for citizens requiring emergency assistance from police, fire brigade or emergency medical services (EMS). 1–1-2 calls are received by three national command centers: two operated by the police and one by the Copenhagen fire brigade, forwarding medical emergencies to the relevant health care regional Emergency Medical Dispatch Center (EMDC). Each EMDC is responsible for the EMS response from receiving the call until the patient is handed over to a hospital or patient contact either has been finalized on scene or during the call. Each health region has its own EMDC that operates prehospital units, using criteria-based dispatch [6]. The Danish EMS has been described in detail elsewhere [7].

The Danish EMS is a three-tiered system, comprising emergency medical technician ambulances, paramedic ambulances and anesthesiologist-staffed mobile emergency care units (MECU) [8]. A nationwide anesthesiologist-staffed helicopter EMS (HEMS) can be dispatched by all five health care regions. In total, approximately 300 ambulances, twenty-six MECU and four HEMS helicopters are available in Denmark. Military medical helicopter assistance can also be provided in MI or harsh, adverse weather conditions.

### Danish crisis management principles

Crisis management in Denmark relies on seven principles that include sector responsibility principle, the cooperation principle and the action principle. The crisis management principles are summarized in Table 1. Danish National Crisis and MI Management System is outlined in Additional file 1.

**Table 1:** Principles applied in Danish crisis management

Principle	Actions
Sector-responsibility principle	Agencies responsible for a similar type of incident in a smaller scale will remain responsible in a major incident
Cooperation principle	Both public services and non-government organizations have a responsibility to cooperate in the rescue effort; i.e. both in the preparedness and incident management phases
Similarity-principle	Organizational structure in major incident management must remain similar to the daily structure
Proximity-principle	Tasks in the major incident management should be undertaken as close as possible to citizens and at the lowest possible organizational level
Flexibility principle	Actions and decisions taken by an major incident authority should be adapted to the current situation, i.e. the task dictates the actions taken
Action principle	In an uncertain situation with insufficient information, major incident management and preparedness should be raised. Every authority is obliged to act
Direction principle	Actions of major incident management should be derived from strategic intentions, e.g. form prepared plans at hand

### Danish trauma system

In a two-tiered system, Danish trauma management includes both regional and university hospitals. Trauma referral centers are located in Copenhagen, Odense, Aarhus and Aalborg. They all provide definitive care for 500 000 to 2 800 000 people since catchment areas differ between the five regions. Some trauma centers have national competencies, such as a burns unit, hyperbaric oxygen treatment, limb saving surgery etc.

### Major incident preparedness

In Denmark, a set of guidelines for joint services incident command, [9] constitutes a theoretical and practical framework for MI management. The concept accounts for every aspect of interdisciplinary MI management and incident commanders are trained during a three-week, joint service course offered by the Danish Emergency Management Agency. During the course, several table-top and full-scale exercises are conducted and comprehensive training in the use of communication devices is included. After completion of the course and passing a final examination, Police, Fire & Rescue, and Medical Incident Commanders share a common language and understanding of MI management.

### Communication

In Denmark, authorities responsible for safety, health, and public order utilize a nationwide secure emergency radio network, a Terrestrial Trunked Radio [10] TETRA standard based system. According to the national guidelines for joint services incident command [9, 11], the police issue a temporary interdisciplinary communication channel within the TETRA-based system in the event of an incident involving multiple authorities. EMS units are expected to switch radios to the assigned Health sector channel, as forced steering or patching of communication channels in MI is not yet in use in Denmark [12]. Incident Commanders have a dedicated channel for internal, joint service command communication.

### Copenhagen emergency medical services

EMS in the Copenhagen metropolitan area is operated by the Capital Region. The EMDC is located in Ballerup and features a 24/7 in-house coordinating senior prehospital physician with the overall medical responsibility for the EMS-response in the region. Up until 62 ambulances operate from 21 ambulance stations around the region. Five MECUs are on duty from four bases, three of these 24/7. The HEMS helicopters from Danish Air Ambulance are available for the Capital Region as well. Positioned strategically across Denmark, one of these helicopters is located on a base in Ringsted, 50 km south-west of Copenhagen. The Capital region also operates two mobile Emergency Room trailers/casualty clearing stations which may be mobilized in MI (See Additional file 2).

### Tactical emergency casualty care

Tactical emergency casualty care, TECC®, is a concept for training EMS personnel on how to respond to and care for patients in a civilian tactical environment [13]. The TECC® concept focuses on situational awareness and treatment in the safe zone of a tactical environment and does not include personal protective equipment.

### Tactical emergency medical service

TEMS is a concept to ensure that certified and specially equipped paramedics and prehospital physicians are able to enter an area that is not yet declared safe by police to perform triage and time critical lifesaving emergency procedures to stop patients dying from e.g., gunshot wounds, stabbing etc. The TEMS teams are educated and trained in working in a tactical setting on not yet secured scenes. They pass a demanding physical test every year and train tactically on a regular basis with the police. A TEMS team is on duty 24/7, staffing one of the five MECUs in the Capital Region, carrying their personal protective gear and equipment for MI.

TEMS has been operational in the Capital Region since 2018 [14]. The TEMS unit is dispatched to approximately 200 incidents per year, ranging from assistance to the police arresting known dangerous perpetrators or assessing potentially violent psychotic patients to actual or threatening terrorist incidents. TEMS is seconded to the police as needed and is under police command and protection when deployed. Between TEMS tasks, the team functions as a standard MECU at the disposal of the EMDC.

### Copenhagen hospitals

**Fig. 1:**
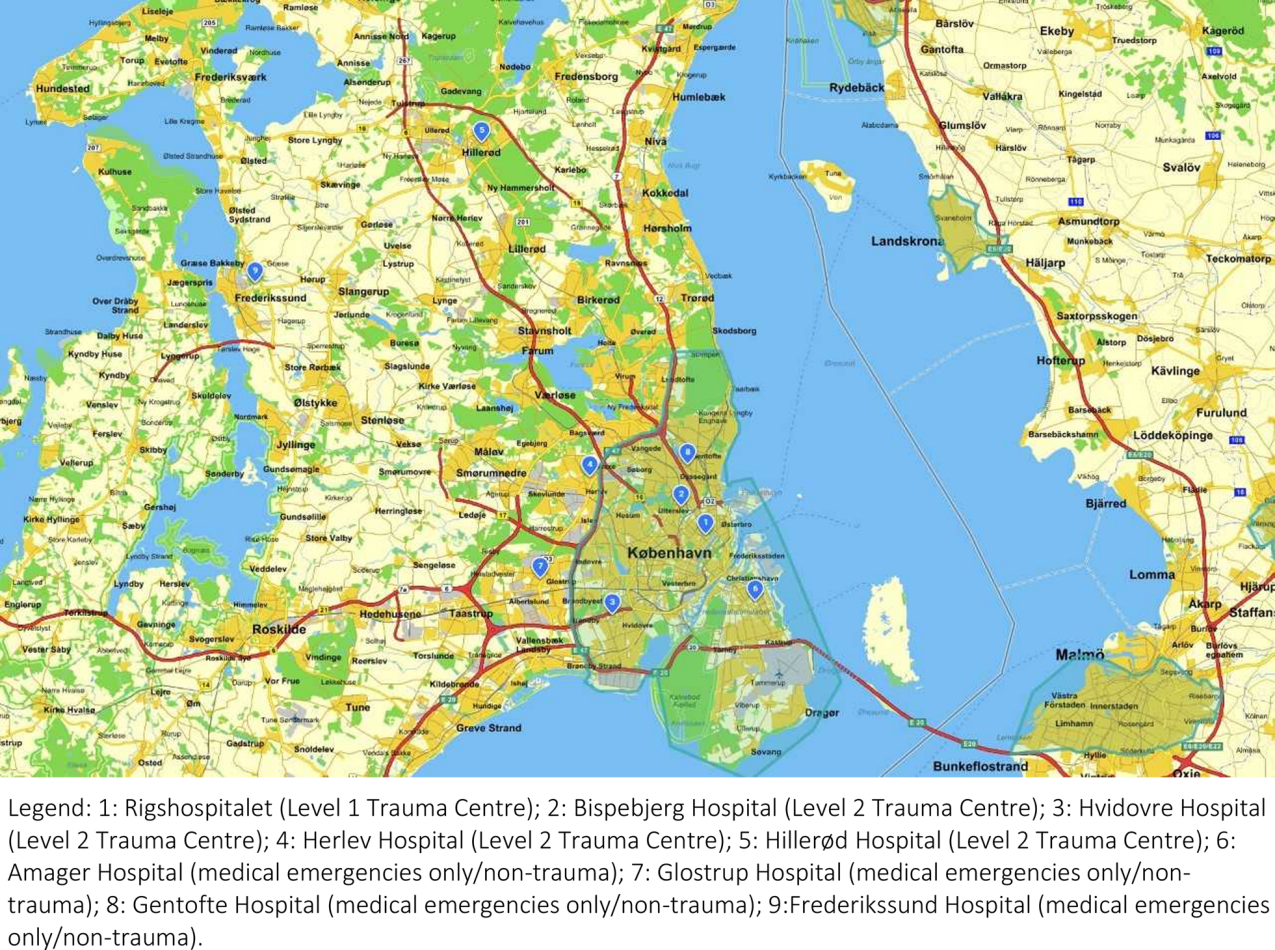
Copenhagen metropolitan area hospitals

There are five emergency university hospitals in the Copenhagen metropolitan area, located in Hvidovre, Herlev, Bispebjerg, Hillerød and Copenhagen City where Rigshospitalet, a Level 1 trauma centre with a catchment population of 2 800 000 people, is situated. In addition, four smaller hospitals are part of the MI preparedness plans with the capability to treat lightly injured patients (See Fig. 1).

### Scene description

Field’s is one of Denmark’s largest shopping malls. It comprises 135 stores, nine cinemas and twenty-two restaurants. Situated seven kilometers from the center of Copenhagen with a nearby metro station, the 115 000 square meters mall is popular among families and young people. An estimated 15–40 000 people visit the mall on a regular day. In the event of concerts in the nearby Royal Arena, a 16 000 capacity indoor sports and concert venue, it is customary that Field’s restaurants are used extensively prior to the event.

### Study design

The present case report describes the prehospital EMS response on 3rd July, 2022 to the mass shooting at Field’s, Copenhagen, Denmark. The case report adheres to the CONFIDE (CONsensus guidelines on Reports of Field Observations in Disasters and Emergencies) [15] concept, used in the assessment of the quality of non-traditional studies, intended to acquire the optimal evidence approach to MI and disaster response. (See Additional file 3).

### Data acquisition

Data sources included:

- Control room system LOGIS® (Nærum, Denmark), Copenhagen EMS, Ballerup, Denmark
- The electronic Prehospital patient medical record system (Judex®, Aalborg, Denmark)
- Center of Emergency Communication, Frederiksberg, Copenhagen, Denmark
- Public domain

### Alarm and dispatch

The national distress number 1–1-2 received the first of over 550 calls from the incident site at 17.33. Copenhagen Police dispatched units to the incident at 17:35. Copenhagen EMS was alerted at 17:39. The call taker in the EMDC acknowledged that the incident was potentially serious and initiated dispatch of a MECU as the Medical Incident Commander (MIC) and the first ambulance at 17.42. Radio contact was established between MIC, Police, and Fire Brigade Incident Commanders en route and a contact point 200 m from the mall main entrance was agreed upon.

Upon arrival as the first medical unit on scene at 17:49, simultaneously with the Fire Incident Commander (FIC), immediate physical contact was made with the Police Incident Commander (PIC) and Joint Incident Command (JIC) was established. The exact incident location was verified, and the presence of active shooter(s) was confirmed. One shooter had just been apprehended, and one or two more gunmen were still believed to be at large in the mall. Four severely injured in need of immediate treatment were being evacuated from inside the mall by police. Based upon the size of the mall, the assumed number of assailants and number of visitors, a joint assessment of a potential double-digit number of casualties was agreed on. MIC reported immediately back to the EMDC, formally declaring MI at 17:50.

### Site access and security

**Fig. 2:**
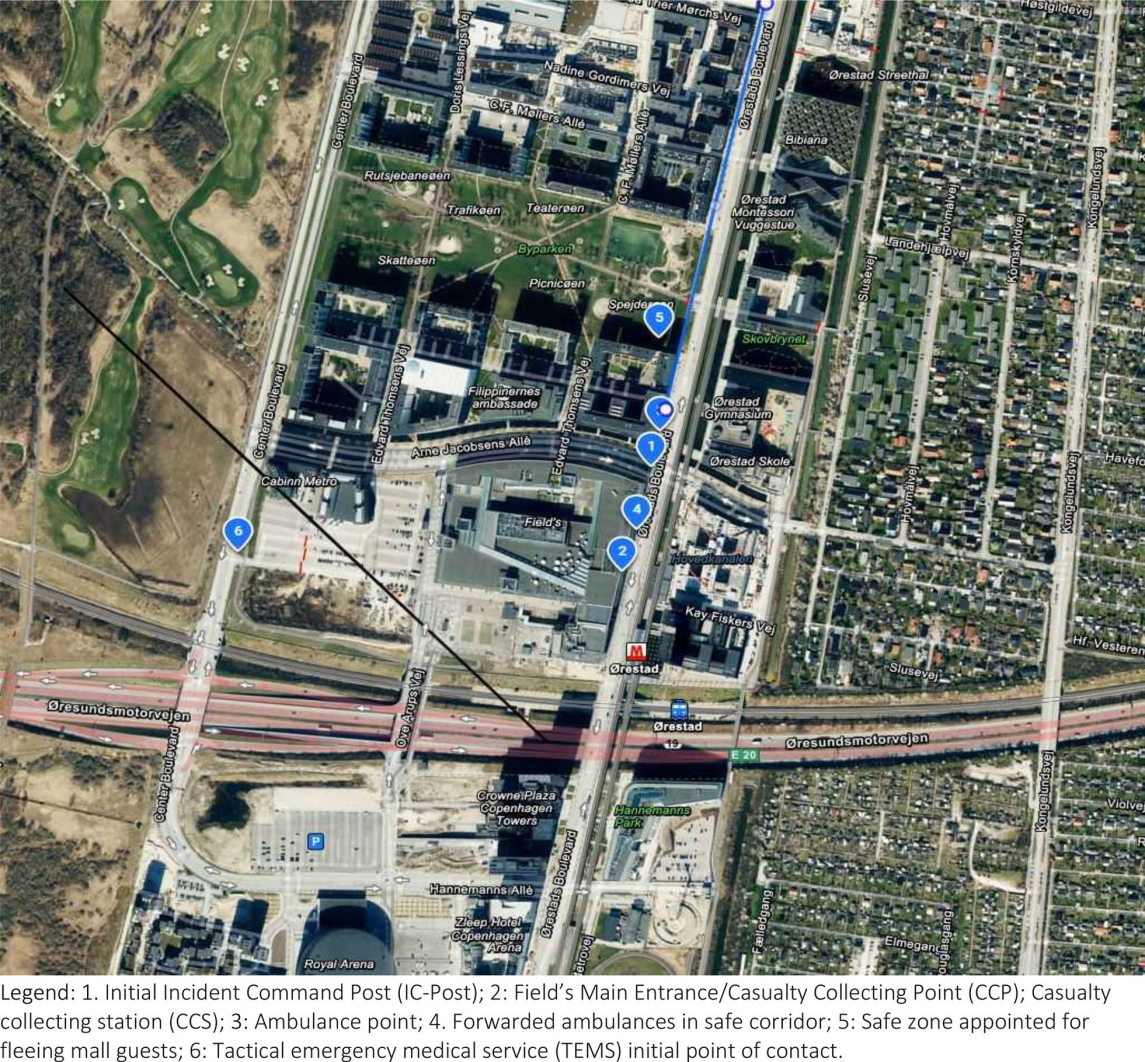
Field’s incident site organization

The mall has multiple entry points, including the main entrance on the eastern side of the complex, additional entrances and access points from in-house parking lots. MIC designated an ambulance staging area approximately 200 m north of the main entrance (See Fig. 2), with safe access/egress via a specific ambulance route from the north. The staging area was not in direct line of potential fire from within the mall.

A massive presence of armed police formed a secure corridor from the ambulance point to the main entrance. This protected passage was established within minutes after being requested by MIC and was in place before the second ambulance arrived on scene. The corridor enabled a coordinated and safe evacuation by ambulance of injured people from an interim Casualty Collection Point (CCP) established by tactical fire units at street level outside the main entrance. Close and ongoing liaison between incident commanders was maintained throughout the initial critical phase.

Arriving EMS units were, initially individually and later in groups, regularly briefed by MIC on the situation, including safety precautions.

Mall guests fleeing the incident via the main entrance were herded by police and directed away from the scene. The first sight that met MIC approaching the scene was a huge crowd of people running in panic toward the blue lights of the MECU. Having passed a city park immediately before arriving at the Incident Command contact point, MIC directed the crowd to continue running towards the park where they could assemble. The park itself was completely shielded from the mall by a tall housing complex and deemed to be safe. It was already noted at this point that some individuals were lightly injured, i.e., walking wounded.

The traffic on the Metro railway line opposite Field´s Mall was halted and the station, surrounding streets and junctions as well as the nearby Royal Arena were secured by armed police.

### Site organization

The incident site was organized as per guidelines and dynamically adapted according to the rapidly developing scenario. The crew comprising physician and paramedic/physician’s assistant from the first MECU dispatched to the scene continued in the roles of MIC and Medical Communications Officer (MCO) for the duration.

Extensive inner and outer cordons were established by police according to the nature of the incident and the ensuing manhunt for multiple perpetrators presumed still at large. The danger zone consisted initially of the entire inside of the mall, gradually being reduced in size as police incrementally swept, cleared, and secured segments of the building.

A Joint Services Incident Command Post (IC-Post), physically consisting of a MECU and the Fire Incident Commander command vehicles parked next to each other, was set up at the road junction at the northeast corner of the mall (See Fig. 2). The location provided an excellent visual overview of the safe corridor established by armed police to the south, along the eastern façade towards the main entrance and CCP, as well as north towards the ambulance staging area and access/egress route. A spacious and well-staffed and equipped Police Incident Command module was set up on the west side of the mall to lead police operations. Normally serving as a Joint Incident Command post, the module was more than 600 m away from the focus of medical operations and its facilities were thus unavailable for MIC.

An interim CCP was designated at the foot of the stairs leading up to the main entrance of the mall. It was decided by the joint incident command that neither a Casualty Evacuation Point (CEP) nor a Casualty Clearing Station (CCS) for secondary triage could be established close to the mall in the chaos phase of the incident due to safety issues and lack of personnel. It was therefore decided that ambulances would be called forward as needed through the safe corridor to load patients at the CCP after primary triage. Treatment would be provided en route to the hospital according to the load-and-go principle. The second MECU on-scene was deployed to the CCP as Forward Medical Commander in order to supervise triage and report back to MIC. A manoeuvre plan for setting up a CCS indoors in a secure location on the ground floor of the mall close to the main entrance if needed, once safety had been assured, was agreed on by incident commanders.

Standard key roles such as Ambulance Commander, Ambulance Loading Officer, ambulance personnel for staffing CCS and Casualty Clearing Officer were intentionally not designated due to a critical lack of resources in the initial phase, where all ambulances were needed for immediate transport. See Fig. 2 for site organization.

### TETRA communication

65.3% of the EMS units switched to the designated interdisciplinary talk group. Seventy TETRA radios were in use in the MI. There were 213 radio grid related shifts. The 34.7% that did not switch as intended primarily switched by mistake to the joint incident command channel instead of the designated HEALTH channel or had trouble shifting the radios. There were no reports of compromised TETRA network bands coverage difficulties.

Strict radio discipline was enforced from an early stage by MIC/MCO, keeping assigned and designated channels clear of unnecessary communication. All EMS units were issued a default listen only order on the common, designated HEALTH channel. In acknowledgement of the inherent different perspectives on the situation as it unfolded, MIC and EMDC communicated continuously on an assigned EMDC channel, with regular updates reported up the chain of command from the scene and operational lead and support provided from the EMDC. Other key roles could participate on the EMDC channel by invitation. An example of this was the Trauma Centre at Rigshospitalet, which provided invaluable information and regular updates regarding surge capacity status.

TEMS teams reported status and findings to MIC/MCO at intervals over a dedicated tactical channel on a *do not answer* basis, verifying or refuting rumors regarding the number and type of casualties and deceased. The Forward Medical Officer and the mobile medical teams, consisting of on-duty MECUs and mobilized off-duty MECU staff, were assigned a common, dedicated channel for medical coordination at the scene and reporting back to MIC. The Incident Commanders from the three sectors remained in contact by radio when not physically together.

### Ambulance resources

Ten ambulances, four on-duty MECUs and a mobile emergency room trailer (MERT) were requested immediately by MIC as well as the mobilization of further ambulances as and when they became available. Of the first ten ambulances requested, five were intended as per MI protocol for command support roles and for preparing a Casualty Clearing Station (CCS)/MERT, while the other five ambulances were intended for transport of priority 1 casualties. Furthermore, assistance from the neighboring Region Zealand and mobilization of off-duty prehospital personnel from home were advocated. Safe access/egress along a specific route from the north with ambulance parking north and out of sight of the mall was ordered. The on-duty TEMS team was notified directly by Copenhagen police, arriving in a MECU on the west side of the mall at 17:57, and was immediately deployed under police command and protection.

The first ambulance arrived at the incident at 17:53, three minutes after MI was declared.

The next three ambulances arrived 14, 21 and 24 min after the first ambulance. A second MECU arrived at 18.17 together with the fourth ambulance.

**Fig. 3:**
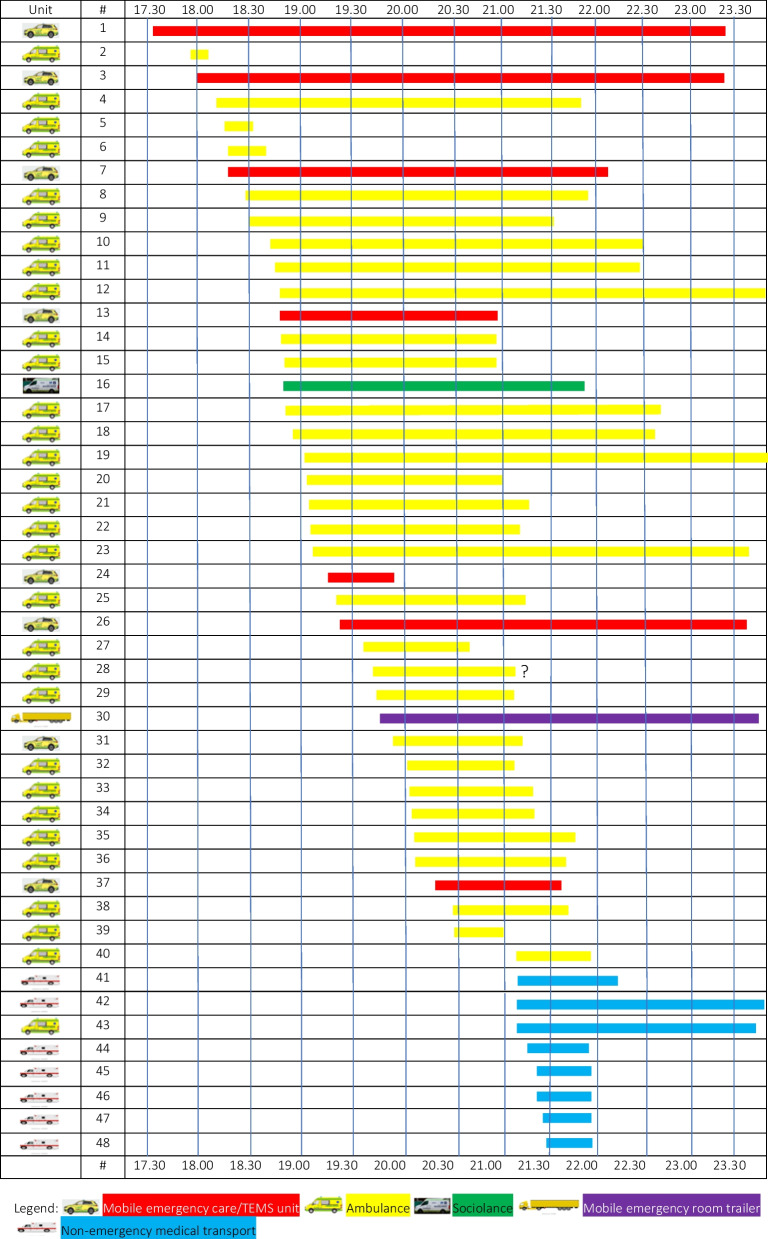
Graphic presentation of dispatched units and time expenditure

In total, 48 EMS units were dispatched sequentially during the entire incident, including 31 ambulances, eight MECUs (five of these as TEMS teams), seven Non-Emergency Medical Transport (NEMT) vehicles, and social services mobile unit, and one mobile emergency room trailer (See Table 2 and Fig. 3). Off-duty personnel, including four TEMS teams using off-duty MECU vehicles for transportation, were called in from home, issued with uniforms and kit at the EMDC Major Incident Preparedness depot, teamed up and transported successively to the scene.

**Table 2:** Table of dispatched units

Unit #	Unit name	Type	Arrival	Departure
1	L01	MECU 1	17.49	23.27
2	R03	Ambulance	17.53	18.09
3	L09	TEMS 1	17.57	23.27
4	A91	Ambulance	18.07	22.07
5	A77	Ambulance	18.14	18.33
6	A98	Ambulance	18.17	18.41
7	L06	MECU 2	18.17	22.15
8	A35	Ambulance	18.27	22.05
9	A05	Ambulance	18.29	21.40
10	A85	Ambulance	18.38	22.40
11	A83	Ambulance	18.39	22.37
12	A32	Ambulance	18.42	00.12
13	L03	MECU 3	18.42	21.16
14	R03	Ambulance	18.43	21.16
15	A77	Ambulance	18.44	21.16
16	S05	Sociolance	18.45	22.05
17	A90	Ambulance	18.46	22.47
18	A23	Ambulance	18.50	22.40
19	A74	Ambulance	18.59	00.19
20	A97	Ambulance	19.00	21.04
21	A38	Ambulance	19.01	21.18
22	A43	Ambulance	19.01	21.07
23	O04TNB	Ambulance	19.03	23.27
24	L14	TEMS 2	19.09	19.43
25	A89	Ambulance	19.10	21.12
26	L11	TEMS 3	19.18	23.27
27	A88	Ambulance	19.30	20.35
28	O03VES	Ambulance	19.34	?
29	S02	PCU	19.35	21.01
30	S13	MERT	19.36	23.27
31	L04	TEMS 4	19.46	21.07
32	A09	Ambulance	19.58	21.01
33	A22	Ambulance	19.59	21.22
34	A75	Ambulance	20.00	21.24
35	O01HIL	Ambulance	20.01	21.55
36	A49	Ambulance	20.01	21.49
37	L08	TEMS 5	20.14	21.40
38	A17	Ambulance	20.28	21.48
39	A34	Ambulance	20.28	20.52
40	R01	Ambulance	21.05	21.50
41	T32	NEMT	21.05	22.11
42	T28	NEMT	21.15	23.47
43	A33	Ambulance	21.15	23.34
44	T06	NEMT	21.23	22.03
45	4707	NEMT	21.31	22.04
46	4710	NEMT	21.31	22.05
47	T18	NEMT	21.34	22.03
48	4708	NEMT	21.37	22.05

MECU: Mobile emergency care unit; Tactical emergency medical service; PCU: Psychiatric care unit; MERT: mobile emergency room trailer; NEMT: non-emergency medical transport

To avoid crowding of ambulances at the incident site and to ensure capacity for regular ambulance services, EMDC decided to position eleven ambulances at five ambulance bases and assembly points within a 5 km radius of Field’s for possible rapid dispatch to the incident site. Furthermore, in order to maintain regular ambulance services in Copenhagen, EMDC commissioned ambulances from the neighboring region for possible ordinary missions besides the MI. An overview of the available ambulances is provided in Table 3.

**Table 3:** Overview of available emergency medical services unit availability

Timestamp	Ambulances available	Ambulances en route	MECU available
17.42	18	2	3
17.50	17	1	1
17.55	17	1	1
18.00	17	0	1
18.05	17	0	1
18.10	18	0	2
18.15	18	0	2
18.20	20	0	2
18.25	23	0	2
18.30	23	0	1
18.35	27	0	1
18.50	26	0	1
19.00	25	1	3

MECU: Mobile emergency care unit

### Patient treatment and characteristics

All ambulance transported patients were treated en route to the hospital. Treatment consisted primarily of lifesaving first aid measures, i.e., tourniquets/wound packing/hemorrhage control as warranted at the CCP, supplemented with oxygen, large bore intravenous cannula, intravenous fluid, and pain medication with opioids as needed during transport. All four critical patients had verified or assumed thoraco-abdominal gunshot wounds and were in varying degrees of circulatory shock.

### Triage

Initial *eyeballing* triage was undertaken by police and firefighters at the CCP after evacuation of wounded from the danger zone. Three victims were pronounced dead by deployed tactical units during the initial sweep and left on scene in the mall. One severely wounded victim with time critical and immediately life-threatening injuries was evacuated and transported to the trauma center on the rear seat of a police patrol car as ambulances had not yet arrived. Three other evacuated, critically injured patients were triaged by the first ambulance arriving at the scene and reported to MIC over the radio. Triage was performed on the basis of wound location and the Triage Sieve, and all three victims were categorized Priority 1. Transport priority and destination was decided by MIC and a load-and-go order was issued. The remaining casualties were transported at intervals as and when the next two ambulances arrived.

### Tactical emergency medical service

Five TEMS teams were dispatched to the incident site. The on-duty team arrived within minutes, whereas four off-duty teams were mobilized from home, arriving at intervals during the ensuing 90 min. Initially shortly briefed by MIC on arrival, mobilized TEMS teams were immediately seconded to the police and deployed. During this phase of the incident, a manhunt was still in progress, as it was uncertain whether the arrested perpetrator was acting alone, or if more gunmen were on the loose. Teamed up with armed police, TEMS’ primary task was to help search the mall sector by sector and floor by floor for casualties and guests hiding in cinemas, shops, storage rooms, washrooms, stairwells and elevators. TEMS pronounced life-extinct victims dead and left them as they were found for further police investigation.

### Management of uninjured survivors

Local municipal authorities established a Survivor Reception Centre on request from the police at a nearby sports arena, approximately one km from the incident. Uninjured survivors and witnesses were registered and questioned by the police, and psychological support and counseling was provided by a dedicated team of psychologists and psychiatrists.

### A major contributing event in an adjacent arena

In the nearby Royal Arena, a concert featuring British pop star Harry Styles was scheduled to commence at 19.00. The 16 000-capacity venue was about half full at the time of the mass shooting at 17.33. The promoters first postponed the concert to 20.00 but a final decision to cancel the concert was made at 21.37. As per police orders, the Copenhagen Metro was commissioned at 21.41 for transportation of approximately 6000 predominantly teenage concert guests away from the area to a metro station on the other side of the city for safety issues and to avoid inadvertent contamination of the crime scene.

### Continued assessment

As the hours passed, repeated sweeps of the complex revealed no further gunmen or serious casualties in the mall. Several uninjured survivors were found hiding in restrooms, storage rooms, back offices and shuttered shops and were evacuated as and when they were found well into the night. In the meantime, a substantial force of EMS units and personnel had been accumulated. Following briefing, they were initially tasked with setting up a CCS and preparing for secondary triage, treatment and transport of any potential casualties found during the ongoing sweeping operation.

At approximately 20.30, Incident Command acknowledged that any untreated, critically wounded not yet found would already have died from their injuries. A massive presence of EMS was no longer justified, and a majority of units were released from the scene after defusing. A stand down of the mobilized off-duty staff and ambulance crews at the end of their shifts were declared. On-duty units were released for service as needed elsewhere in the region. A de-escalation recommendation was forwarded up the chain of command by MIC, and the hospitals started winding down from 21.30, returning to normal service at 23.00.

All TEMS teams, one MECU, and five ambulances were retained on site with MIC in order to receive and treat any unexpected civilian or tactical casualties until MI was declared closed for EMS around midnight.

### Defusing and debriefing

Fit for the task, a brief on-scene defusing was performed by MIC immediately following the incident before units were released. The ambulance crews were also defused locally, i.e., each ambulance base conducted ad hoc debriefing as per request and guideline. Furthermore, all EMDC personnel and ambulance personnel were offered structured defusing a few hours after the incident or the following day.

Structured debriefings for involved personnel were conducted by Copenhagen EMS as per guidelines for joint services incident command. The debriefings were performed at two meetings, approximately three weeks after the incident took place.

## Discussion

### Challenges encountered by EMS in the Field’s mall mass shooting

#### Site access and security

The entire mall and surrounding streets were in complete lockdown, with armed police in increasing numbers guarding all entrances to the mall as well as all junctions and roads providing access to the mall and its immediate surroundings. After initial confusion regarding the access route, all deployed units arrived by the designated access/egress route from the north (See Fig. 3).

### TETRA communication

Radio communication within EMS and between authorities mainly took place according to predefined radio grid [16]. However, 34.7% of the radio shifts were non-standard. Factors such as lost situation awareness due to unintuitive interface design [17], the startle effect [18] and insufficient basic training in operating communication devices have been previously described in a MI case report [12] and in a survey among Danish prehospital physicians [19].

### Ambulance resources

Ambulance shortage is inherent in MI [20, 21]. In most MI case reports, the need for ambulance resources is most prominent in the initial phase, where casualties with time-critical injuries need immediate transport to definitive care in order to survive. During this incident, all patients in need were transported promptly. Unharmed and lightly injured survivors tend to be able to evacuate themselves, even from an unsafe incident site, simply fleeing from the scene. At the Field’s mall mass shooting, this was also the case.

Since ambulance availability was lacking in the initial uncompensated phase of the incident, MIC was prepared to have the first arriving ambulances each carry two priority 1 patients on a load-and-go basis as well as utilizing police vehicles and fire trucks for time-critical transportation [22, 23]. This is beyond guidelines; yet reflects the need for adaptation, improvisation, and quick decision-making based on the available tools and, as in this case, proximity to a number of University Hospitals. At Fields, non-certified transportation was used in order to ensure timely hospital treatment and survival of one critically injured victim.

Fortunately, the number of presented priority 1 casualties did not warrant further improvised transportation, yet the dilemma and decisions made reflect the call for action principle in crisis management. Also, considering available initial resources, it was decided to postpone the designation of Ambulance Commander, Ambulance Loading Officer, Casualty Clearing Officer and ambulance personnel for staffing the CCS. These considerations reflected the immediate need for the transportation of patients with time-critical injuries weighed against expected time of arrival of further ambulances.

Along with the considerations made above, ambulance availability was also a priority issue for the EMDC. In MI, ambulances already dispatched on non-related urgent and/or lower priority missions, but without a patient onboard, should be immediately re-prioritized to the MI, which was echoed in the dispatch acuity in the Field's mall mass shooting.

A critical decision rests with the EMDC on whether to assign all available ambulances to the incident or hold some back in case of multiple-site, time-staggered incidents and for tasks unrelated to the MI. This demands extremely close and ongoing liaison with police authorities. However, the initial critical phase of a MI demands a minimum response of ambulances to enable triage, lifesaving treatment and establishing a command structure at the scene. Contingency planning for prompt provision of a predefined sufficient ambulance response in MI should be undertaken beforehand and trained at regular intervals at the EMDC.

### EMDC perspective

As it is often the case in MI, the perspective in the EMDC may differ somewhat from that in the field. This is inherently due to the EMDC’s increasingly broader overview of the situation as time progresses, by way of ongoing access to multiple sources of information. Though sharing perspective with field personnel is of high priority, the overall picture will ultimately be more concise in the control room compared to on the ground.

In the initial chaos phase, it was a challenge predicting the need for ambulances, weighed against considerations on reserving resources for possible secondary site attacks as well as everyday emergency events. Also, tactical considerations were undertaken for securing fast ambulance delivery in the event that an access route was cut off and to mitigate a “sitting duck-effect” in an ambulance line-up if perpetrators were to target EMS.

Regional Crisis Management was activated simultaneously with recognition of MI. EMDC coordinated hospital capacity and overall patient allocation with all hospitals in the Capital Region in preparation of a large number of casualties. EMDC also had ongoing communication and coordination with Police and Fire Brigade command centres throughout the incident.

### Ambiguous reports

Status reports were relayed at intervals to Incident Command through different channels from tactical units deployed in the mall regarding the number of casualties and deceased. Numbers varied between five and eight critically wounded casualties, and uncertainty reigned for an extended period as to whether the relayed information concerned victims already accounted for, or if new casualties were being evacuated to the CCP.

Accordingly, MIC forwarded a MECU and five ambulances to a holding position in close proximity to the CCP within the safe corridor provided by the police. The intention was to perform rapid initial triage, treatment and immediate transport from the scene to relevant hospitals as soon as the reported wounded were evacuated from the mall.

When it was finally confirmed that no further casualties were imminently expected at the CCP the IC-Post was relocated to the main entrance of the mall. In the meanwhile, an incremental build-up of EMS units had taken place at the designated staging area. Arriving MECUs and mobilized off-duty medical teams were briefed by MIC and tasked with preparing a CCS while ambulances and NEMT vehicles remained on standby in the staging area.

### EMS mass casualty medical preparedness

The Copenhagen EMS had specifically prepared for an immediate and flexible response to MI and multiple site terrorist attacks for several years. Strategic preparedness planning, crisis management, cooperation and coordination both within the health sector and at all levels with other authorities had been established and trained prior to 3rd July 2022.

An EMS command structure analogous to *Gold—Silver—Bronze* or *Strategic – Operational – Tactical* levels [9] has been an integral part of Danish EMS organization for a more than a decade, with experienced medical commanders at all levels on-site 24/7 (See Additional file 1).

All prehospital emergency physicians in the Capital Region are consultant anesthesiologists, employed at university hospital departments in the region. Mutual agreements between EMS and hospitals ensure dedicated mobilization of off-duty physicians to EMS in the event of MI.

Two Mobile Emergency Room Trailers consisting of a fully equipped emergency room as well as two 40 m^2^ high-pressure, rapid deployable inflatable tents per trailer, were devised, funded and organized for dispatch within 15 min to compensate for initial lack of ambulances in complex and/or multiple site MI in an outdoor setting or adverse environments. Each trailer carries 30 collapsible, NATO standard wheeled gurneys for transport over long distances or rugged, debris-ridden terrain. Furthermore, oxygen, vital-signs monitors, and extensive amounts of medical kit are available in wall-mounted bags. Casualties can thus be treated and stabilized in a protected indoor environment until sufficient ambulance transport is available.

Although requested immediately in connection with declaration of MI, the Mobile Emergency Room Trailer did not arrive until 19:36 for undisclosed reasons. However, by that time, transportation capacity was more than sufficient, and the trailer was not needed. A secondary potential function as a collecting point for unharmed evacuees was also superfluous at the time of arrival.

Standardized Mass Casualty bags, each containing equipment and medication including anesthetics, tranexamic acid and freeze-dried plasma, for treatment of up to 30 casualties (blunt and penetrating trauma, burns, explosions) were designed, packed and stored at the EMDC Major Incident Preparedness depot. MI bags would either be delivered to the scene by blue-light units or collected by mobilized personnel reporting to the depot before deployment. The MI bags were intended for use by medical teams at multiple sites or distributed as necessary at different locations within a single site setting, including the Mobile Emergency Room Trailer. Medical equipment and medication from MECUs and ambulances are quickly depleted in a MI situation but are planned replenished from MI bags arriving within a short space of time.

An often-neglected subject in forward planning, but a major issue during prolonged MI, is the capability of sustaining personnel with food and refreshments, rest and other personal needs and relief from duty in the field. No formal system or plan was in place for sustenance or relieving deployed EMS personnel, some of whom had been working without a break for hours before the incident. EMS staff were without logistical support and at the grateful mercy of their counterparts from the police and fire brigade. Though this may cause few problems in incidents of short duration, EMS organizations should take this into account when planning for prolonged incidents.

### Patient treatment and characteristics

After the evacuation from the danger zone, the patient flow away from the CCP for primary triage and treatment before transport was severely compromised by the ambulance shortage in the early phase of the MI. One of these patients, a patient with a time-critical life-threatening injury, was transported in a police patrol car before the first ambulance arrived. The last severely injured patient was transported from the scene by ambulance roughly an hour after being wounded.

The nature and dynamics of the incident are mirrored in the fact that many patients fled the scene in the initial phase and once the means to establish a CCS were available, the CCS was no longer needed. In this incident, the relatively low number of priority 1 patients compensated for the lack of ambulances. The remaining survivors were directed to safety in a nearby park and presented themselves at hospitals if needed on their own accord.

A loss of overall control of patient flow presents a potential threat to hospital preparedness, as specialized hospitals may be flooded by lightly injured patients. It has previously been described that nearby smaller, non-emergency hospitals may be overrun with moderately or even severely injured patients presenting themselves [24–26].

### Hospital preparedness

When MI was declared, the five emergency hospitals in Copenhagen metropolitan area executed preparedness plans, mobilizing more than 500 hospital employees. In spite of being in the middle of the summer holiday, a substantive number of staff volunteered their assistance. Therefore, hospital resources were sufficient and timely coordinated with the potential arrival of numerous patients from the incident site. The importance of relaying information from incident sites through EMDC to receiving hospitals is pivotal and has been underlined in reports from similar MI [24–26]. Therefore, hospital preparedness plans are of major importance in MI, described in previous literature [27, 28].

### Major incident reporting

As the importance of purveying lessons learned in MI is pivotal to EMS organizations, [29] portals such as the majorincidentreporting.net website, developed by the Major Incident Reporting Collaborators [30] are paramount to future MI management. The portal features a template for reporting details of the EMS response to MI to enhance the sharing of experience and lessons learned from real-life scenarios for future MI management improvement.

The efforts to mitigate the effects of delayed or lost information [29] are important, since information often stem from non-indexed literature such as government hearings [31, 32] and commissions. [33, 34] However, reporting MIs should take into consideration that not all MIs are the results of natural disasters but may be planned and intended to harm society. Thus, the potential exposure of weak spots in the preparedness should be considered.

### Triage

A pragmatic and easy to use regional trauma referral guideline is used on a daily basis by MECU and ambulance staff in the Capital Region, incorporating the capabilities of the different emergency hospitals and round the clock availability of specialists in relevant fields. In penetrating trauma, a load and go principle, with treatment en route is recommended. In MI situations in the Capital Region, the Triage Sieve [35] is advocated as a supplement to anatomical localization of injuries/involved organ systems with regard to decision making.

Triage Sieve [35] is one of many triage systems used in MI [36] and until recently, no triage system has been proven to be significantly superior to another [37]. However, research by Malik et al. [38] found the Battlefield Casualty Drills (BCD) Triage Sieve to outperform all existing MI triage tools and is the preferred triage system in United Kingdom. Considerations of two separate triage systems for blunt and penetrating injuries respectively, might be of relevance.

### Social media

In the hours after the mass shooting, social media insistently purveyed rumors of additional perpetrators. Police surveillance of social media confirmed the circulation of videos of the perpetrator, giving rise to suspicion of associations with religious or ideological extremist organizations. Therefore, the police were not able to declare the scene safe before meticulous search inside the mall had been performed. The action in the Field’s mall was prolonged substantively as the result of rumors and delayed the return to a normal state.

In similar incidents, social media has played a role [39–41]. In a paper by Pan et al. [42], the use of witness sensors in epidemic outbreaks was introduced. The purpose is to mitigate information flaws in MI, and to enhance the spread of facts instead of fiction in critical events. Witness sensors are certified volunteers with basic, intermediate, or expert training in reporting facts from MI on social media under the control of authorities, such as police EMS, Fire & Rescue, etc.

### Tactical emergency medical services performance

Five TEMS teams were dispatched to the scene and were actively engaged in the MI as requested. The TEMS concept in the Copenhagen metropolitan area dates back to 2018 [14] echoing the events at a lone terrorist attack in Copenhagen in 2015 resulting in the killing of two people. The TEMS concept has been active in Finland since 1998 [43], fueled by the Rauma, Jokela [4] and Kauhajoki [44] school shootings and in Norway, the PLIVO concept [45] has been adapted in the EMS after the Oslo/Utøya attacks [3].

As it turned out, the perpetrator was acting on his own, but uncertainty and social media rumors about multiple shooters justified TEMS involvement in the Field's mass shooting. In Europe, several tactical concepts have been developed in the post 9/11 era, including France [46], Spain [47] and in Germany [31, 48]. Similar to MI in general, TEMS is challenged with very low frequency, reflecting the need for micro and full-scale training with joint authorities.

### Lessons learned

- Radio communication should be addressed regularly in training to eliminate erroneous channel settings. Forced steering of radios should be considered.
- The MIC should be equipped with logistical or physical command support and/or a dedicated mobile command module.
- The guidelines for MI should include considerations on improvised transportation in the form of police vehicles, civilian vehicles or other means of rapid transportation. By addressing this matter in guidelines, any MIC should be encouraged to consider these transportation options.
- Contingency planning for prompt provision of a predefined sufficient ambulance response in MI should be undertaken beforehand and trained at regular intervals at the EMDC.
- As is often the case in the initial phase of a MI, the need for ambulances exceeds the availability. The EMS should be prepared to improvise under such circumstances. This may include both considerations with regards to the number of patients transported in each ambulance and the potential for confiscating other privately owned vehicles with the aim of hyper-acute patient transport to the hospitals
- Mobile emergency room/casualty clearing stations should be organized in such a fashion that they can be dispatched immediately for MI and be easily deployable within minutes of arrival.
- Logistical planning concerning the sustainment of EMS personnel with food and refreshments is imperative.
- Witness sensors acting on social media may be introduced to control for erroneous information.

### Strengths and limitations of the study

Data consistency and the availability of all relevant data from encrypted platforms constitute the strength of this case report. It provides a nearly complete picture of all relevant operational details of the EMS response to a MI.

Several limitations are present in this case report, such as selection bias that is inherent in observational studies since a causal relationship between EMS response and patient outcome cannot be established. Accordingly, information bias and confounding exist. Recall and selection bias may have possibly affected the result interpretation. Three of the authors were directly involved in the MI management in prominent, first-line roles, introducing the risk of recall bias. However, the contribution to an almost complete picture of the actual events and operational decisions in the MI is very likely to be present.

The findings of the case report are believed to be generalizable and transferable to prehospital critical care organizations in similar socio-economic and geopolitical arenas.

## Conclusions

The EMS response to the mass shooting at Field's in Copenhagen on 3rd July, 2022 was massive from a Scandinavian perspective. A total of 48 EMS units were dispatched to the scene, involving multiple authorities in the first large-scale mass shooting incident in Denmark. Overall successful EMS performance was the result of a combination of substantial preparedness planning, regular training, and a robust MI management concept. The EMS performance was executed in accordance with preexisting national guidelines and demonstrated that capacity, resilience, and preparedness were substantive.

Important findings included inherent resource shortage that mandated improvised mitigating actions from the Medical Incident Commander in the initial phase. Mono-disciplinary communication shortcomings and the successful use of the tactical emergency medical service concept were also important aspects. Ongoing micro- and macro training between emergency authorities is pivotal for future major incident management to be successful.

### Supplementary Information

**Additional file 1.** Danish National Crisis and Major Incident Management System (from Hansen et al.: The Great Belt train accident: the emergency medical services response).

**Additional file 2.** Mobile emergency room trailer (private photos).

**Additional file 3.** CONFIDE checklist. Quality assessment framework of non-traditional study type.

## Data Availability

The datasets generated and/or analyzed during the current study are available in the Zenodo® repository: https://doi.org/10.5281/zenodo.7902673.

## Abbreviations

CCP: Casualty collecting point
CCS: Casualty clearing station
CSCATTT: Command and control, safety, communication, assessment, triage, treatment, transport
EMDC: Emergency medical dispatch center
EMS: Emergency medical service
FIC: Fire incident commander
IC-Post: Incident Command Post
MECU: Physician staffed mobile emergency care unit.
MI: Major incident
MIC: Medical incident commander
NEMT: Non-emergency medical transport
PIC: Police incident commander
TECC: Tactical emergency casualty care
TEMS: Tactical emergency medical service
TETRA: Terrestrial trunked radio
