# Supplementary material for: The Field’s mass shooting: emergency medical services response
Source: Scand J Trauma Resusc Emerg Med. 2023 Nov 2;31:71. doi: 10.1186/s13049-023-01140-7 (PMC10621148; doi:10.1186/s13049-023-01140-7)
Supplement: Supplementary file 1 — Additional file 1. Danish National Crisis and Major Incident Management System (from Hansen et al.: The Great Belt train accident: the emergency medical services response). [file 13049_2023_1140_MOESM1_ESM.docx]

Additional material 1.

Danish National Crisis and Major Incident Management System (from Hansen et al.: The Great Belt train accident: the emergency medical services response).

Government Security Committee

STRATEGIC LEVEL

Senior Officials Security Committee

National Operational Staff

International Operational Staff

OPERATIONAL LEVEL

Local Operational Staff

**
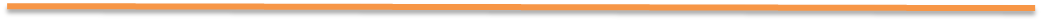
**

TACTICAL LEVEL

Local Incident Command

Police

Fire & Rescue

HEALTH

Emergency Medical Commuication Center

Ambulance Incident Commander

Casualty Clearing Station Officer

Ambulance

SAR/JRCC

HEMS

Ambulance

HEMS: Helicopter Emergency Medical Service; SAR: Search & Rescue; JRCC: Joint Rescue Coordination Center
