# Supplementary figures and images for: The Field’s mass shooting: emergency medical services response
Source: Scand J Trauma Resusc Emerg Med. 2023 Nov 2;31:71. doi: 10.1186/s13049-023-01140-7 (PMC10621148; doi:10.1186/s13049-023-01140-7)

Additional material 2.

Mobile emergency room trailer (private photos).


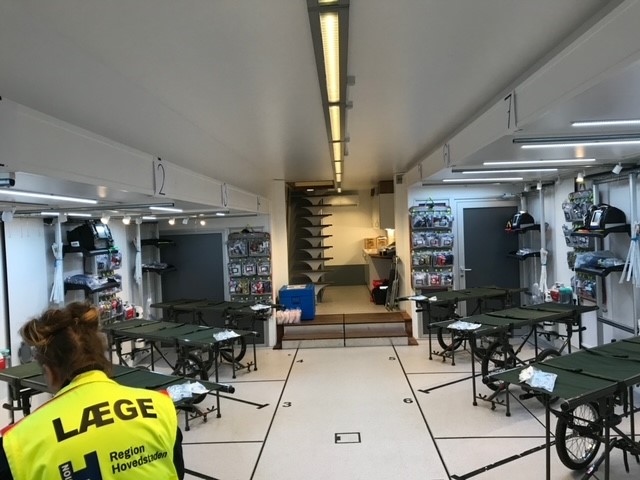


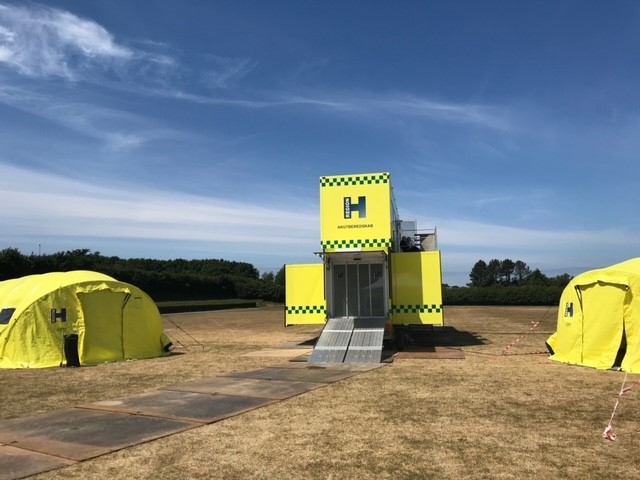


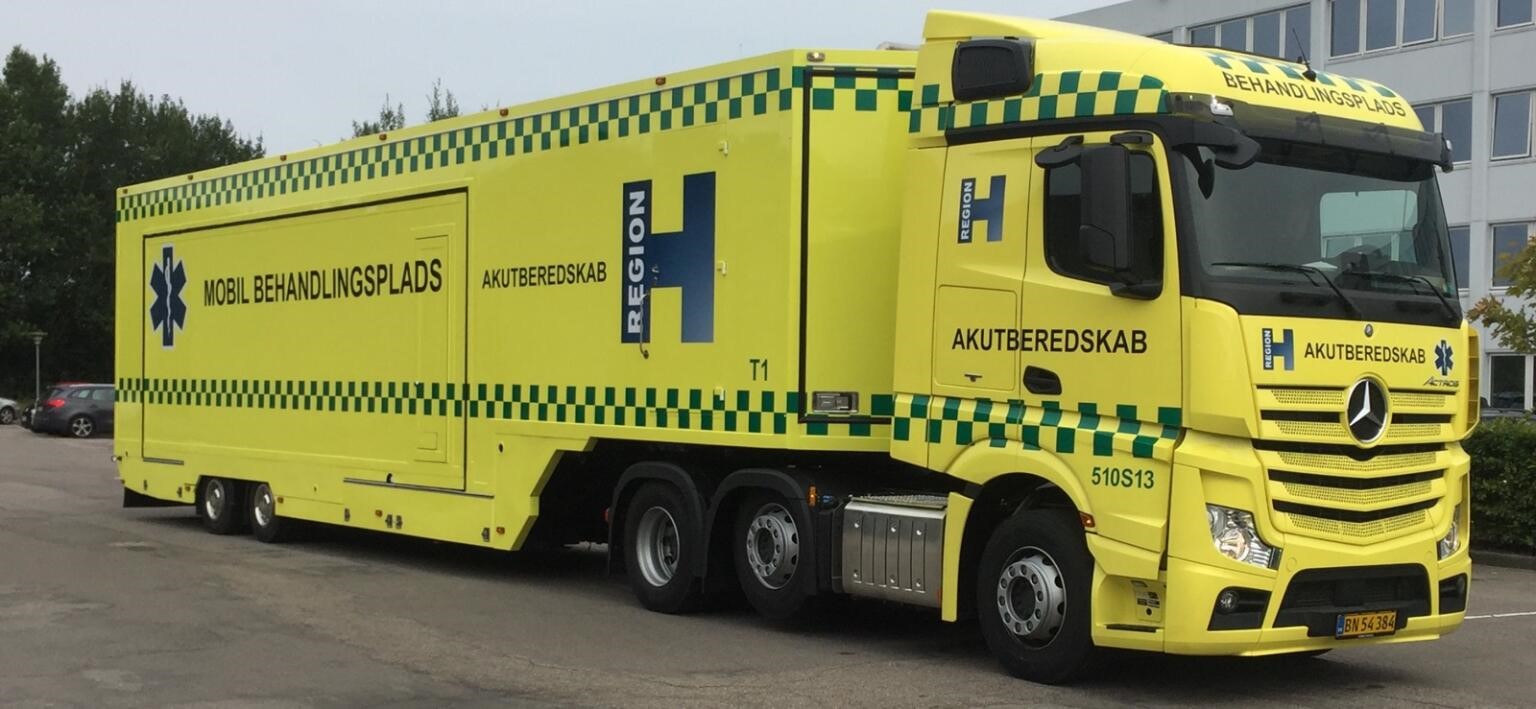

Supplement: Supplementary file 2 — Additional file 2. Mobile emergency room trailer (private photos). [file 13049_2023_1140_MOESM2_ESM.docx]
