# Supplementary material for: The Field’s mass shooting: emergency medical services response
Source: Scand J Trauma Resusc Emerg Med. 2023 Nov 2;31:71. doi: 10.1186/s13049-023-01140-7 (PMC10621148; doi:10.1186/s13049-023-01140-7)
Supplement: Supplementary file 3 — Additional file 3. CONFIDE checklist. Quality assessment framework of non-traditional study type. [file 13049_2023_1140_MOESM3_ESM.docx]

Additional material 3.

CONFIDE checklist. Quality assessment framework of non-traditional study type.

**Robustness: study information and context**

**Score Time period of study/data collection Definition**

A Clearly defined: Exact time frame of data collection given

B Some definition: Approximate time frame given

C No definition: No time frame given

**Score Sample population Definition**

A Clearly defined: 3 out of 4 parameters from age, gender,

number and study population taken from

B Some definition: 1 or 2 parameters defined from above list

C No definition: No parameters defined

N N/A: Not applicable - none population study

**Score Disease description/patient condition Definition**

A Clearly defined

B Some definition

C No definition

N N/A

**Score Author bias Definition**

A Clearly defined Clear who the author is writing on behalf of

with some reflection of potential bias

B Some definition Clear who the author is writing on behalf of

with minimal reflection of potential bias

C None Either not clear who the author is writing behalf of or

clear but with no acknowledgement of potential bias

**Score Type of language Definition**

A Minimal Factual; less than 10% emotive language

B Low bias Less than 30% of language includes below

C Moderate bias 30 - 50% of language includes below

D High bias Greater than 50% emotive language used: use of

"I"; personal beliefs, reflections or experiences;

inner experiences

**Generalizability**

**Score Author perspective Definition**

A The study was written and reported in country by a

native of the country.

B The study was written and reported in country by an

expatriate working as part of the response.

C The study was written and reported externally (to

the country) by an expatriate working as part of or

observing the response.

**Score Applicability Definition**

A Very applicable Results/findings are applicable, relevant and likely

to be similar in other settings

B Moderately applicable Results/findings are applicable, relevant and likely

to be similar in other settings but some aspects are

specific to the study setting only

C Low applicability Results/findings are only applicable and relevant to

the study setting and not likely to be similar in other

settings.

N N/A Opinion piece

**Added value**

**Score Lessons learned Definition**

A High System/organisational level lessons learned,

described and discussed

B Moderate Individual lessons learned

C Low None or limited lessons learned

**Score Triangulation to the literature Definition**

A High Findings linked to previous studies, guidance or

literature as part of discussion

B Moderate Some linkage to previous studies, guidance or

literature

C Low No linkage to previous studies, guidance or

literature

**Score Implications Definition**

A Good Contributes something new and/or different in

terms of understanding/insight or perspective;

suggests ideas for further research; suggests

implications for policy and/or practice

B Fair 1 or 2 of the above

C None None of the above

**Ethics**

**Score Ethics Definition**

A Good Where relevant ethical consideration has been

discussed and approval gained (e.g. in use of photos, patient identifiable data, informed consent)

B Fair Some mention of ethical considerations but no clear

ethical approval sought or gained

C None No mention of ethical consideration; unclear if

permission sought or gained

N N/A Not relevant/none population study
